# The role of long non‐coding RNAs in lung cancer metastasis: Molecular mechanisms, pathogenesis and clinical implications

**DOI:** 10.1002/ctm2.70429

**Published:** 2025-08-10

**Authors:** Musaffe Tuna, Gordon B Mills, Christopher I. Amos

**Affiliations:** ^1^ Department of Medicine Baylor College of Medicine Houston USA; ^2^ Institute of Clinical and Translational Research Baylor College of Medicine Houston USA; ^3^ Division of Oncological Sciences Oregon Health Science University Portland USA; ^4^ Knight Cancer Institute Oregon Health Science University Portland USA

**Keywords:** circular RNA, lncRNA, lung cancer, metastasis, non‐coding RNA

## Abstract

Lung cancer remains the leading cause of cancer‐related death in both women and men in the United States and globally. Even among early‐stage patients who undergo surgical resection, a significant portion of patients develop metastases. Notably, approximately 30% of patients with stage I lung cancer experience relapse. For decades, protein‐coding genes dominated cancer research, driven by the belief that these genes were the primary contributors to tumorigenesis. Despite advances in treatment our understanding of fundamental mechanismsdriving lung cancer progression remains limited. The overall 5‐year survival rate for all stages of lung cancer combined is approximately 20%. Surgical resection remains the best option for early‐stage diseases, whereas chemotherapy, immunotherapy or combination therapies are primarily employed in advanced stages. Frequent treatment failure due to therapy resistance, highlight the urgent need to identify novel biomarkers for early diagnosis, prognosis, and the development of more effective therapies. This underscores the necessity and urgency of researching lncRNAs. The advent of next‐generation sequencing and other high‐throughput technologies guided the discovery of a new class of molecules, non‐coding RNAs (ncRNAs) that play a role in many aspects of cellular physiology. Among the various types of ncRNAs, long non‐coding RNAs (lncRNAs)—which have transcripts longer than 200 nucleotides—have emerged as key regulatory molecules in a myriad of cell functions by interacting with DNA, with other RNAs, including mRNA, miRNA and with proteins. Importantly, lncRNAs play crucial roles in cancer progression, including metastasis by activating oncogenic pathways, promoting epithelial‐mesenchymal transition, remodelling the extracellular matrix, and inducing angiogenesis. Notably, they can function as both oncogenes and tumour suppressors. *MALAT1* is one of the lncRNAs that contribute to metastasis and resistance to tyrosine kinase inhibitors in lung cancer. This review summarizes the role of lncRNAs in cancer, with a specific emphasis on their contributions to lung cancer metastasis.

**Key points:**

- LncRNAs are significant players in lung cancer metastasis.
- LncRNAs can be used as therapeutic target in lung cancer.
- LncRNAs can be used as prognostic factors in lung cancer.
- Exosomal lncRNAs can be used to predict prognosis in lung cancer.

## Graphical abstract

LncRNAs are localized in cytosol, nucleus, other sub‐nuclear compartments, and exosomes in the cell, and play significant role in lung cancer dissemination based on their localizations. LncRNAs have emerged as promising biomarkers for both early detection and prognosis.

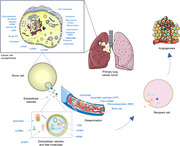

## 1 INTRODUCTION

Lung cancer is the most frequent malignancy about 234 580 people (116 310 men and 118 270 women) predicted to be diagnosed with lung cancer, and about 125 070 people predicted to die from the disease in 2024 in the USA. Worldwide, lung cancer accounts for approximately 2.5 million new cases and 1.8 million deaths in 2020.^1^ Due to the continued high fatality rate, lung cancer remains a universal public health problem. Lung cancer has two main histological subtypes, non‐small cell lung cancer (NSCLC), which accounts for 85% of lung cancer cases, and small cell lung cancer (SCLC).^2^ NSCLC is further sub‐divided into adenocarcinoma (LUAD), squamous cell^3^ and large cell carcinoma.^4^ Despite extensive genomic studies and advances in personalized therapy, the 5‐year survival rate remains at an unfortunate 20% for all stages of lung cancer combined.^5^ The main reason for the abysmal outcome is that patients are usually diagnosed at a late stage and most therapies do not yield durable responses. A significant portion of early‐stage LUAD patients develop lymph node^6^ metastasis and around 30% of early‐stage (stage I) lung cancer patients relapse even with current aggressive therapy.^7^ The frequency of locoregional and distant metastasis formation is dependent on the site of the primary tumour with most cancer deaths being due to metastatic spread^8^, ^9^ with brain being the most common metastatic site (30%).^10^ It is thus crucial to understand the metastatic process and discover biomarkers to develop approaches to predict which patients will metastasize and also to develop effective treatment plans for both local and metastatic disease.

Protein‐coding genes and their role in metastasis have been well studied. However, protein‐coding genes make up only a small proportion (∼ 2%) of the entire human genome. Technological advances and accumulating data show that many noncoding RNAs (ncRNAs), including small, pseudogenes and long noncoding RNAs are present in the remaining part of the genome.^11^, ^12^ Understanding the function of ncRNAs in cancer is just the beginning. An ncRNAs encompass a broad range of known, newly discovered and yet to be identified RNA species. ncRNAs can be classified into two major categories depending on their lengths: small and long ncRNAs. Small ncRNAs include the well‐studied miRNAs, which are usually about 22 nucleotides (nt) long. miRNAs serve as regulators of both protein‐coding and non‐coding genes. In addition to the better characterized miRNAs, small ncRNAs also include many more recently identified and less well‐studied small ncRNAs, such as piwi‐interacting RNAs (piRNAs, 24–31 nt in length)^13^ and small interfering RNAs (siRNAs, 20–22 nt).^14^ It is important to note that while coding RNAs, mRNA, are required for translation and protein expression, ncRNAs such as microRNAs (miRNA), small interfering RNAs (siRNAs), circular RNAs (circRNA), and lncRNAs have critical regulatory roles in target gene expression as well as a myriad of additional functions that are beginning to be elucidated.

This review summarizes the role of lncRNAs in cancer with an initial broad description of the subtypes and potential functions across tumour lineages with a specific emphasis on their contributions to lung cancer metastasis. In terms of each type or subtype of lncRNA, we provide examples of specific lncRNAs that illustrate the defining characteristics of each subclass. We graphically represent in a series of figures the relationships between the different subtypes of lncRNAs. We include a more detailed description of the role of lncRNA in general in lung cancer and a detailed description of several lncRNAs that are involved in lung cancer metastases later in the manuscript.

## 2 Overview of long non‐coding RNAs

**FIGURE 1:**
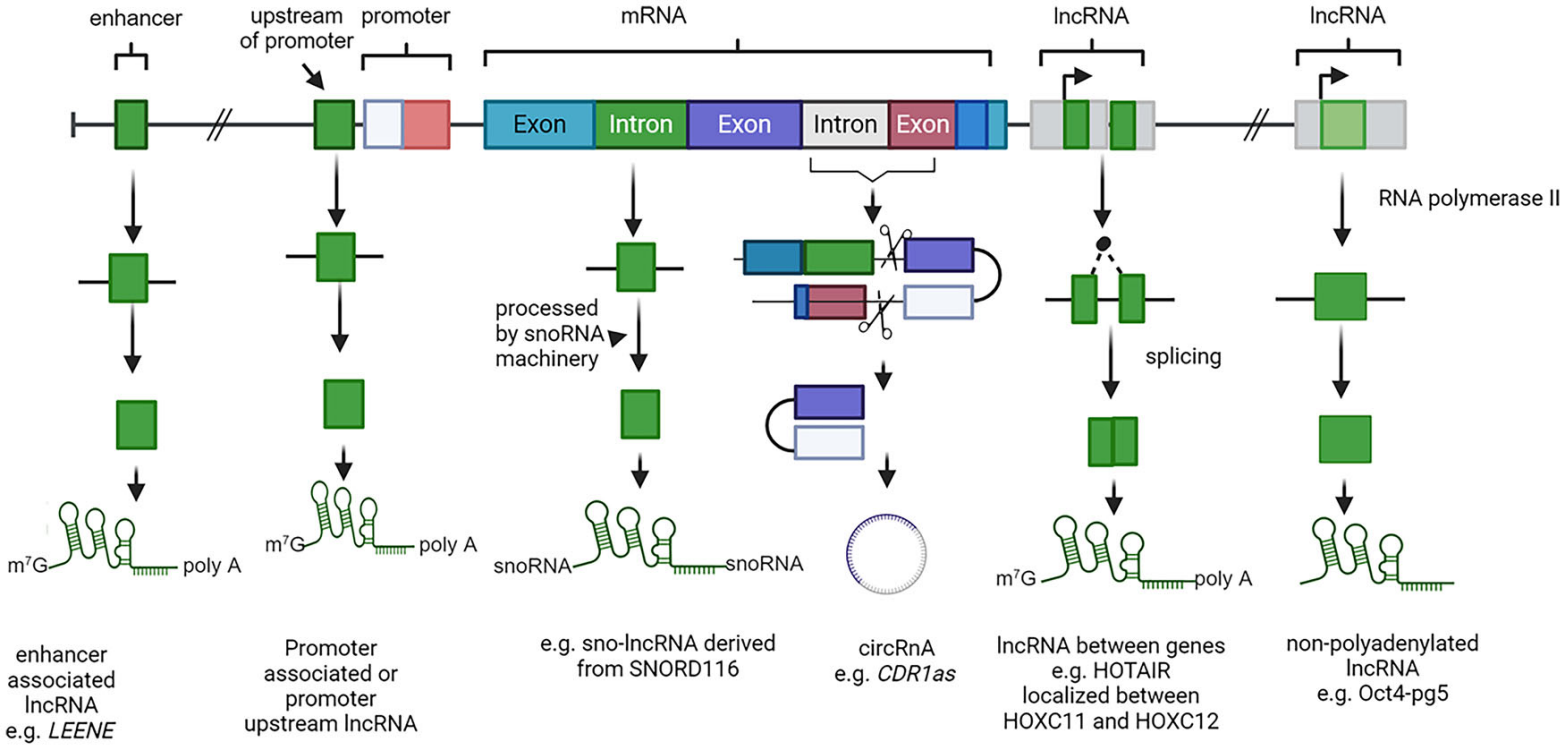
Representative figure showing type of long non‐coding RNAs (lncRNAs). Biorender was used to generate this figure.

In 1989, *H19* was one of the first eukaryote lncRNA reported.^15^ According to the current GENCODE (GRCh38.p14) release v46, there are 20 310 lncRNA genes, 59 927 lncRNA loci transcripts and 7565 small noncoding RNA genes, compared to 19 411 protein‐coding genes and 89 581 protein‐coding transcripts in the human genome (https://www.gencodegenes.org/human/stats_46.html) in 2024, while 15 875 lncRNAs genes and 26 412 transcripts were reported in 2014. Protein‐coding transcripts reflect alternative isoforms of the genes, with, for example, the *BRCA1* gene having 5 protein‐coding transcripts or isoforms. The number of known lncRNA transcripts has grown tremendously in the last decade, indicating that thousands of lncRNAs exist in the human genome, suggesting that there are many more lncRNAs to be discovered and also stressing their significance in cellular biology. Nonetheless, the importance of gene regulation and the function of the vast majority of lncRNAs remain unknown. LncRNAs include circular RNAs (circRNAs; e.g., *CDR1as*),^16^ sno‐lncRNAs (small nucleolar RNA‐derived long noncoding RNAs), SPA‐lncRNAs, ribosomal 18S and 28S RNAs (rRNAs), long or large intergenic ncRNAs (lincRNAs), transcribed ultraconserved regions (T‐UCRs), pseudogenes, GAA‐repeat containing RNAs (GRC‐RNAs), long‐intronic RNAs, promoter‐associated long RNAs (PALRs), promoter upstream transcripts (PROMPTs),^17^ enhancer associated lncRNA (e.g., *LEENE* and *RAIN*) antisense RNAs (aRNAs), stable excised intron RNAs, long stress‐induced non‐coding transcripts (LSINCTs)^18^, ^19^ (Figure 1). lncRNAs have been involved in a variety of biological processes, including cell proliferation, invasion, migration, and epithelial‐to‐mesenchymal transition in cancer cells. The localization and interaction of lncRNAs with other genes and proteins can be variable in different cells and cell lineages, suggesting that lncRNAs have diverse functions. LncRNAs can be separated into broad classes based on their location in the genome, subcellular localization, function, biological states and association with other elements.

The long non‐coding RNAs (lncRNAs) are defined as RNA transcripts that are > 200 nucleotides in length [e.g., *BC200* (Brain cytoplasmic 200 long‐noncoding RNA, 200 nt in length), *XIST* (X inactive specific transcript, 19 kb in length)], with the vast majority not containing open reading frames (ORFs) and not having translated components. A small minority of lncRNAs can contain one or more ORFs, which can be translated into functional small peptides (< 100 amino acids in length).^20^ Previously, these peptides were considered as noise or false positives.^21^ ORF containing lncRNAs include lnc *UBAP1‐AST6*,^22^ *HOXB‐AS3*,^23^ and SRA (steroid receptor RNA activator, 0.87 kb in length) that can be translated into a protein that acts as an RNA coactivator for the steroid receptor complex, Vitamin D and MyoD receptors.^24^ Most lncRNAs are transcribed by RNA polymerase II (Pol II), and linear lncRNAs contain 5′m7G caps and 3′ poly(A) tails and are spliced similarly to mRNA (Figure 1). However, some functional lncRNAs are not polyadenylated [e.g., *asOct4‐pg5* (antisense to Oct4 pseudogene 5) or *BC200*, also known as *BCYRN1*].^25^, ^26^ Some lncRNAs can produce new RNA species with unique transcription patterns like sno‐lncRNAs (e.g., sno‐lncRNA derived from SNORD116)^27^ and 5′ small nucleolar RNA (snoRNA) capped and 3′ polyadenylated lncRNA (SPA lncRNA). Sno‐lncRNAs are a diverse group of non‐coding RNAs that represent a class of long noncoding RNAs that are characterized by their nuclear localization and the fact that they are derived from introns. These RNAs are processed by the small nucleolar RNA (snoRNA) machinery at both their 5′ and 3′ ends.^27^ Sno‐lncRNAs serve either in the modification of snRNAs or rRNA or are involved in the processing of rRNA during ribosome subunit maturation.^28^ Sno‐lncRNAs have snoRNA caps at both ends, whereas SPA‐lncRNAs have snoRNA caps only 5′ end and are polyadenylated in the 3’. Sno‐lncRNAs represent a group of long non‐coding RNAs that share certain structural and functional features with snoRNAs, which have roles in RNA modification (such as rRNA methylation and pseudouridylation). Sno‐lncRNAs can guide the modification or processing of other RNA species, including those involved in splicing or the stability of other non‐coding RNAs, and appear to control the regulation of translation and ribosome biogenesis, which are key processes in cell growth and stress responses.^27^, ^29^ LncRNAs that are processed by ribonuclease P can result in the formation of tRNA‐like structures with increased stability in the cytoplasm (e.g., *MALAT1*, metastasis‐associated lung adenocarcinoma transcript 1 and *NEAT1*, nuclear enriched abundant transcript 1).^30^, ^31^ Compared to mRNA, most lncRNAs contain fewer exons^32^ and in contrast to mRNAs that tend to be expressed in multiple tissues, 29% of ncRNAs are tissue specific.^33^, ^34^

## 3 Classification of lncRNAs

### 3.1 Classification based on genomic localization and function

**FIGURE 2:**
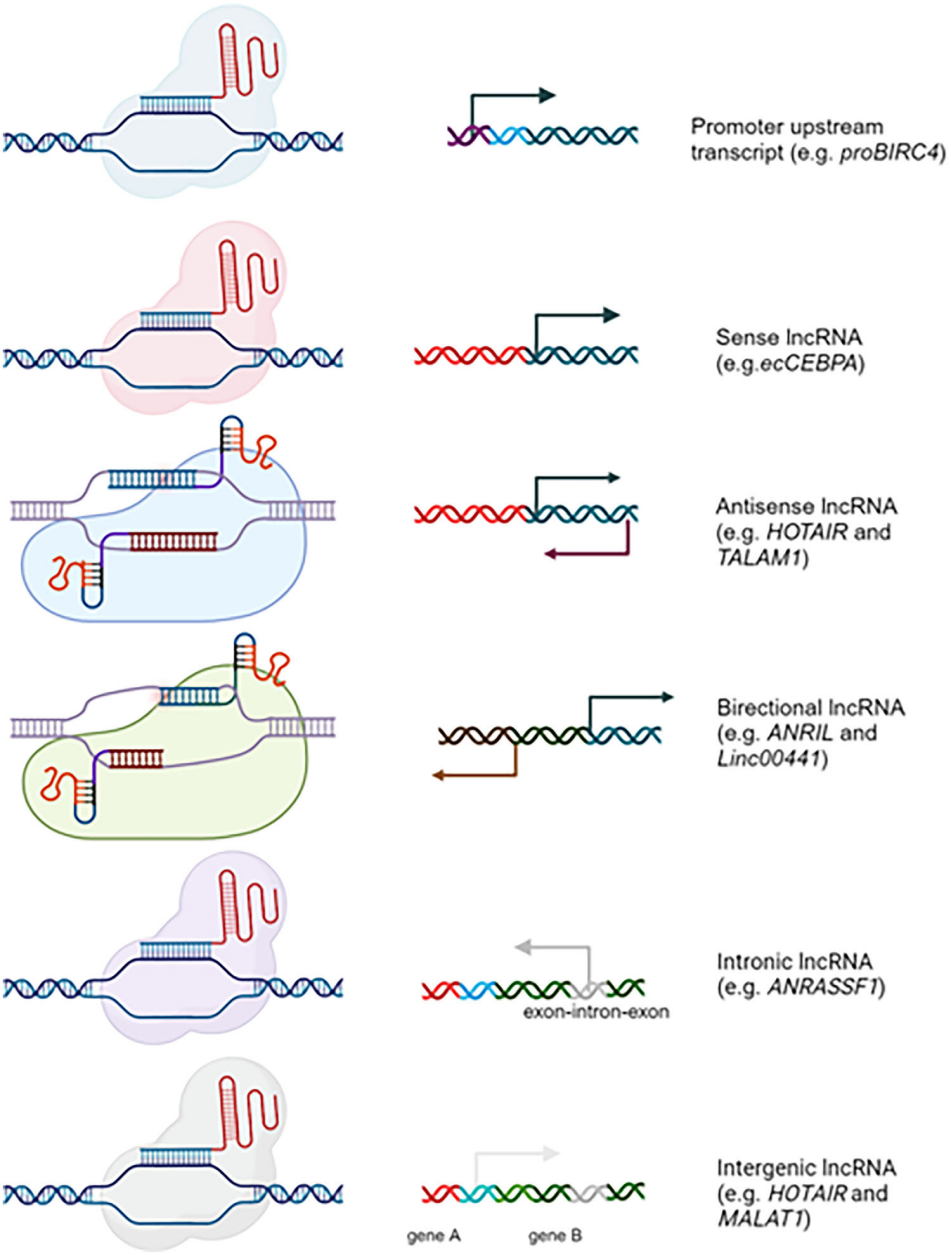
Representative figure showing long non‐coding RNAs (lncRNAs) based on their genomic location. Biorender was used to generate this figure.

lncRNA can be classified into 5 classes based on proximity to neighbouring protein‐coding genes and transcriptional orientation including (Figure 2): (1) Functional DNA element regions: promoter upstream transcript (PROMPTs) (e.g., *proBIRC4*),^35^ enhancer‐associated RNA (eRNAs) (e.g., *EVF2* and *LEENE*),^36^ promoter‐associated long RNAs (PALRs) (e.g., *FOXCUT*).^37^, ^38^ (2) Coding regions: sense lncRNAs (e.g., CCAAT/ enhancer binding protein alpha—*ecCEBPA*),^39^ and antisense lncRNAs that overlap and are transcribed from the same or opposite strand of ORF genes (e.g., HOX transcript antisense RNA, *HOTAIR*),^40^ and (3) intergenic lncRNA (e.g., lncRNA *MALAT1* and antisense lncRNA *TALAM1*),^41^ (4) bidirectional lncRNAs, typically transcribed from both sense and antisense of its neighbouring protein‐coding gene (e.g., *ANRIL, exon 1 of ANRIL is* mapped between the promoter of ARF and INKB4).^42^ (5) Noncoding regions: Intronic lncRNAs (e.g., *ANRASSF1*)^43^ are transcribed entirely from introns, and (5) intergenic lncRNAs (lincRNAs) (e.g., *HOTAIR* and *MALAT1*, also called *NEAT2*, nuclear‐enriched abundant transcript 2). The colocalization of lncRNAs with protein‐coding genes could contribute to the coregulation of expression with the nearby protein‐coding gene or could play a role in regulating expression of the collocated protein‐coding gene. Indeed as an example, some lncRNAs are localized to genomically imprinted regions such as H19 (mapped at 11p15.5), *XIST* (mapped at Xq13 within X‐inactivation centre), and sno‐lncRNA derived from SNORD116 (localized at chromosome 15q11.2‐q13, the Prader Willi syndrome region), *Ube3a‐ATS* (mapped at 15q11.2‐q13), *Airn* (mapped in the second intron of the Igfr2 gene at 6q25.3), *Kcnq1ot1* (mapped in KCNQ1 gene at 11p15.5), and *Meg3* (14q32.3).^42^ Some of the lncRNAs carry binding sites for transcription factors with for example, the SNORD116‐derived sno‐lncRNA containing multiple Fox2 binding sites^27^, ^44^ that could either act as transcription factor sinks or alternatively contribute to the regulation of the nearby protein‐coding gene. In an initially unexpected role, imprinted lncRNAs have been proposed to lead to allele‐specific silencing of genes where they are localized.^44^ This suggests that the genomic localization of lncRNA could regulate genomic imprinting, allele‐based methylation of genes, with these processes potentially playing a role in selective pressures driving the localization of a subset of lncRNAs.

### 3.2 Classification based on subcellular localization

**FIGURE 3:**
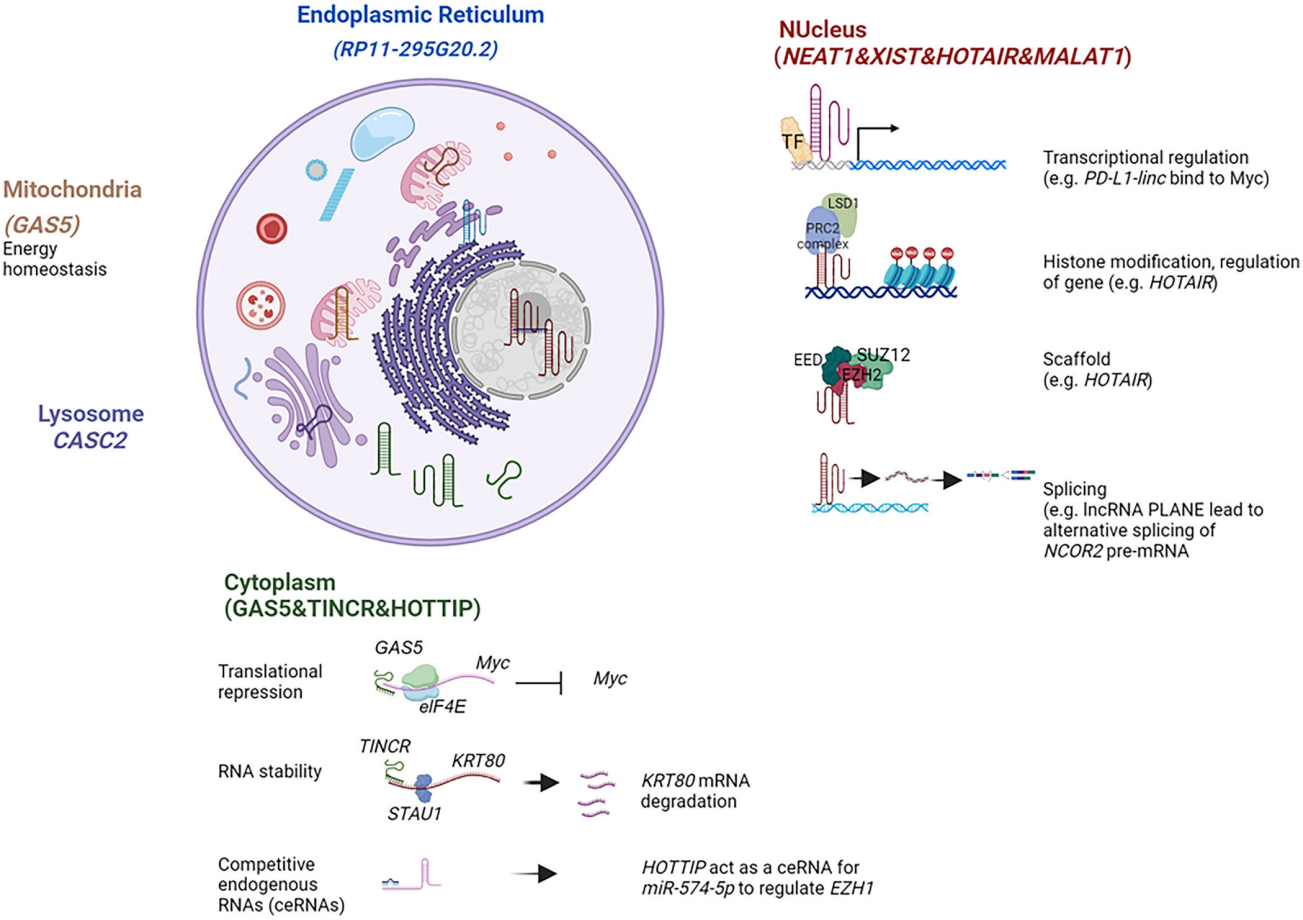
Representative figure showing long non‐coding RNAs (lncRNAs) based on subcellular localization. Biorender was used to generate this figure.

Subcellular localization of lncRNAs plays a critical role in the functions of different lncRNAs, providing an alternative lncRNA classification. The majority of lncRNAs are localized in three sub‐nuclear compartments (chromatin, nucleolus and nucleoplasm)^39^, ^45^, ^46^ where their major roles appear to be in regulating gene transcription^47^ (Figure 3). Many lncRNAs localize to chromatin where they interact with nuclear proteins, including transcription factors to promote or inhibit their binding and activity at targeted DNA regions. lncRNAs can bind and recruit epigenetic modifiers to specific loci, providing additional levels of transcriptional control. lncRNAs can also localize to other nuclear domains such as nuclear speckles (e.g., *MALAT1* and *NEAT1*)^48^, ^49^, ^50^ where their functions remain fully defined, with some being described in more detail below. In addition, a number of lncRNA are dominantly localized in the cytosol where they exert their functions through forming complexes with RNA and RNA‐binding proteins, altering RNA processing, mRNA stability, or translational efficiency or through direct regulation of the function of a number of proteins.^51^ Cytosolic lncRNA can change localization to fulfil their function with for example, the *PAPAS* lncRNA that is normally present in mitochondria in the cytosol, relocalizing into the nucleus in response to cellular stress to mitigate stress‐induced damage.^52^ Further, lncRNA can localize to cytosolic organelles including ribosomes (e.g., *GAS5 and ZFAS1*),^53^, ^54^ mitochondria (e.g., *HITT* (HIF‐1a inhibitor at translational level, also known as *LINC00637*), endoplasmic reticulum stress‐associated (e.g., *RP11‐295G20.2*),^55^ lysosome (e.g., *CASC2*)^56^ and exosomes and other organelles.^46^, ^57^ Mitochondria‐localized lncRNAs can be encoded by nuclear (e.g., *AFAP1‐AS1*)^58^ or mitochondrial DNA (e.g., *MDL1AS*, *lncND5*, and *ASncmt*)^59^ and have been related to mitochondrial metabolism, cell growth and apoptosis.^60^, ^61^ For example, *GAS5* (growth‐arrest‐specific 5) binds to malate dehydrogenase 2 to regulate cellular energy homeostasis through the mitochondrial tricarboxylic acid cycle, with mitochondrial metabolism being crucial to tumour initiation and progression.^56^, ^62^ *ZFAS1* interacts with the small 40S subunit of the ribosome. In an emerging and critical function, lncRNAs can be incorporated into exosomes and other extracellular vesicles where they can transit to recipient cells and alter the function of the recipient cells, including epigenetic regulation, cell‐type reprogramming, and regulation of genomic stability.^63^

### 3.3 Based on function

In addition to localization, llncRNAs can be classified based on their cellular functions (Table 1). These are summarized in Table 1 and include primarily nuclear functions of modulating chromatin remodelling and function, *cis* and *trans* regulation of DNA transcription (e.g., *lncPRESS1*, *NEAT1*, *HOTTIP*),^64^, ^65^, ^66^ replication (e.g., *CONCR*),^67^ response to DNA damage repair (e.g., *GUARDIN*),^68^ genome integrity (e.g., *SCAT7*),^69^ regulating the assembly and function of membrane‐less nuclear bodies (e.g., *NEAT1_2*, long transcript of *NEAT1*),^70^ and genome stability (e.g., *NORAD*, *CCAT2*).^71^, ^72^ Methylated *NEAT1* is involved in the repair of dsDNA breaks.^73^ Moreover, in the cytosol, the primarily control mRNA splicing (e.g., *SPA1*, *SPA2* and lncRNA *BC200*),^29^, ^74^ alters the stability and translation of cytoplasmic mRNAs (e.g., *FOXM1‐AS* and *MEG3*).^75^, ^76^ Through these diverse processes, lncRNA influence innate immunity, regulate protein function or localization (e.g., *SNHG5*),^39^, ^77^ alter chromatin interactions (e.g., *CCAT1‐L*),^39^, ^78^ mediate intercellular communication (e.g., *H19*),^79^ and act as miRNA sponges (e.g., *TP73‐AS1*, *LINC00673*, *LCAT1*).^80^, ^81^, ^82^

**TABLE 1:** Long non‐coding RNAs (lncRNAs) based on their function.

Function	lncRNAs	Reference
DNA transcription	*lncPRESS1*, *NEAT1*, *HOTTIP*	^64^, ^65^, ^66^
DNA replication	*CONCR*	^67^
Response to DNA damage repair	*GUARDIN*	^68^
Genome integrity	*SCAT7*	^69^
Genome stability	*NORAD*, *CCAT2*	^71^, ^72^
RNA splicing	*BC200*	^74^
Regulate the assembly and function of membrane‐less nuclear bodies (acting as architectural scaffolds)	*NEAT1_2* (long isoform of *NEAT1*)	^70^
Alter the stability and translation of cytoplasmic mRNA	*TINCR*	^83^, ^84^
Influence innate immunity	*GAS5*, *NEAT1*	^85^
Regulate protein stability, function or localization	*DINO*, *GUARDIN*	^68^, ^86^
Alter chromatin interaction	*CCAT1‐L*	^39^
Mediate intercellular communication	*H19*	^79^
Act as competitive endogenous RNA	*TP73‐AS1*, *LINC000673*, *LCAT1*	^80^, ^81^, ^82^

#### 3.3.1 The function of lncRNAs in cis

The expression of neighbouring genes can be regulated (cis‐regulation) by lncRNA through multiple mechanisms: (1) LncRNAs have emerged as key regulators of gene expression, especially in the context of neighbouring genes. These lncRNAs can influence the expression of adjacent genes by interacting with various regulatory factors and modulating their function, such as *lincRNA‐p21*.^87^ (2) Transcriptional or splicing‐dependent regulation of neighbouring genes can be independent of the specific RNA sequence.^88^ For example, an imprinted lncRNA RNA derived from the antisense of the parental alleles of the imprinted IGF2R gene, *Airn*, overlaps with the IGF2R gene and its promoter. It is essential for silencing the paternal allele by recruiting silencing factors leading to a transcriptionally repressive chromatin state.^89^ *Airn* leads to histone modifications such as H3K9me3 (a mark of heterochromatin), and DNA methylation at the promoter region of IGF2R, which contribute to transcriptional silencing of the paternal allele. (3) Cis‐regulation of gene expression also depends on functional DNA elements within lncRNA loci. For example, *lincRNA‐p21* is classified as an enhancer RNA that is associated with the regulation of CDKN1A.^90^

#### 3.3.2 The function of lncRNAs in trans

LncRNAs can regulate chromatin states and gene expression at regions distant from their transcription sites by acting as a scaffold and coordinating the recruitment of chromatin‐modifying complexes to regulatory sites in the distant gene (e.g., *HOTAIR, MALAT1*). *HOTAIR* induces epigenetic regulation by binding to the histone H3 lysine 27 (H3K27) methylase complex, mammalian polycomb repressive complex 2 (PRC2), and histone demethylase complex, lysine‐specific demethylase 1 (LSD1), and broadly regulates gene expression.^91^, ^92^ *MALAT1* is recruited to nuclear speckles through direct interactions with multiple‐associated proteins and regulates alternative splicing of multiple different RNAs.^93^, ^94^, ^95^ Overexpression of Colon Cancer Associated Transcript 2 (*CCAT2*), which is localized in 8q24.21, can induce chromosomal instability by interacting with and stabilizing the BOP1 ribosomal biogenesis factor, leading to increased MYC expression in colorectal cancer.^71^ Moreover, the *CCAT2* rs6983267 SNP contributes to changes in the secondary structure of the *CCAT2* lncRNA, modulating cellular energy metabolism in an allele‐specific manner.^96^ This SNP is associated with the risk of developing colorectal cancer.^97^

### 3.4 Classification can be based on association with DNA elements

LncRNAs can also be grouped based on association with specific DNA elements: enhancer‐ and promoter‐associated and repeat‐associated lncRNAs. Enhancer‐ and promoter‐associated lncRNAs are transcribed from active enhancers: eRNAs and enhancer‐associated lncRNAs (elncRNAs), where they regulate gene expression. While eRNAs are relatively well‐defined, elncRNAs represent a broader category that includes various long, often polyadenylated RNAs associated with enhancer regions. eRNAs are relatively short in length, bidirectionally transcribed, capped at the 5′ end, not spliced, not polyadenylated, and not stable, whereas elncRNAs are mostly unidirectional, polyadenylated, and spliced, making them more stable in cells than eRNAs.^98^, ^99^ elncRNAs involved in: (1) regulating transcriptional initiation at gene promoters, (2) chromatin remodelling through interactions with chromatin‐modifying complexes, (3) maintaining enhancer‐promoter interactions over longer distances, ensuring proper gene activation and silencing. ElncRNAs are often associated with active enhancer marks such as H3K27ac and H3K4me1, allowing regulation of a broad set of genes. The *HOTTIP* elncRNA, which is transcribed from a distal enhancer region, regulates transcription of the HOXA gene cluster during development and stem cell differentiation.^46^ Overexpression of *HOTTIP* has been associated with worse prognosis in small‐cell lung cancer by regulating EZH2 and BCL‐2, altering differentiation and drug resistance.^100^, ^101^

The human genome contains many repeats, and they frequently overlap with repeat‐associated lncRNAs. The presence of repetitive RNA elements in long non‐coding RNAs (lncRNAs) can be crucial for their sub‐cellular localization, including guiding them to the nucleus (e.g., functional Intergenic Repeat Element [FIRRE]). FIRRE contains multiple repeating RNA domains (RRDs), which are responsible for the ability to interact with specific proteins, such as hnRNPU (heterogeneous nuclear ribonucleoprotein U), that may influence gene transcription or 3D chromatin architecture. This interaction is a key for FIRRE's localization to the chromatin, particularly in the nucleus, where it can play a role in regulating gene expression and chromatin organization.^102^ The RRDs within FIRRE and similar lncRNAs serve as binding sites for proteins that help anchor these RNAs to specific chromatin regions or subnuclear structures, contributing to the regulation of gene expression and chromatin organization.^102^ Other lncRNAs, such as *XIST* and *NEAT1* (which form part of paraspeckles), also utilize repeat‐based interactions for their nuclear localization and function.^46^

This underscores an interesting feature of many lncRNAs: far from being simply “passive” molecules, many of them use specialized domains (often rich in repeats) to interact with the nuclear machinery, guiding their function in chromatin remodelling, gene regulation and other cellular processes. The repetitive elements could also help stabilize these interactions or promote the formation of multivalent complexes that facilitate the function of lncRNAs.^46^

In addition, many lncRNAs are composed of sequences derived from transposable elements (TE), or they contain portions that are highly homologous to transposons that enable their interactions with specific chromatin regions, RNA‐binding proteins, or other molecules involved in cellular regulation. An example, *XIST* contains ERVB5‐originating TE repeats that recruit SPEN (transcriptional repressor) to enforce inactivation of the X chromosome.^103^, ^104^ Transposable elements can also regulate lncRNA expression through TE‐derived promoters that are activated during development and differentiation, as well as in response to cellular stress. *HOTAIR* contains intronic regions with features of retrotransposons and is regulated by multiple cellular processes.^105^ Mutation or loss in TE within lncRNA can disturb their function with for example, the SINEB, a specific type of SINE (short interspersed nuclear element), element located at the 5′ end of *MALAT1*, regulating its nuclear localization and its function. When the SINEB1 element is deleted, the localization of *MALAT1* is disrupted, resulting in cytoplasmic accumulation instead of remaining in the nucleus, disrupting the nuclear functions of *MALAT1*, leading to chromosomal instability and mitotic catastrophe.

In summary, lncRNAs exhibit complex and diverse structural and functional characteristics. Their various classifications—based on genomic localization, subcellular distribution, function, or association with DNA elements—highlights their remarkable diversity from different perspectives. These distinctive features endow lncRNAs to play crucial roles in a wide range of cellular biological processes. As a result, they provide a fundamental basis for further research into their roles in diseases such as cancer, laying a solid foundation for further exploration in this field.

## 4 Current state of lncRNA in cancer

Dysregulation of lncRNAs has been identified in numerous diseases, including cancer. Based on the functions described above, lncRNA can play multiple roles in the initiation and progression of cancer, including response to therapy. Due to the complexity of the functions of lncRNA, different lncRNA have characteristics that are compatible with oncogenic or tumour suppressor function. The effects of the lncRNA can also be contextual, depending on other processes going on in the cells.

### 4.1 Genetic and epigenetic deregulation of lncRNAs in cancer including lung cancer

LncRNAs can be dysregulated by multiple mechanisms including mutations, structural alterations such as amplification or translocation, as well as changes in expression or stability. *LINC00662*, which is at a region that is often amplified in NSCLC, epigenetically regulates the BIK viability regulator through interacting with EZH2.^106^ Another amplified lncRNA, *PVT1* (plasmacytoma variant translocation, regulates VEGFC (vascular endothelial growth factor C) by acting as a ceRNA for miR‐128 in lung cancer with elevated expression being associated with poor survival.^107^ *SNHG6* and SNHG17 are amplified in NSCLC^108^, ^109^ contributing to migration and proliferation. *PLANE* is upregulated through amplification as well as E2F1‐mediated transcriptional activation.^110^

*MALAT1*, which spans the *Alpha* locus at 11q13.1, is overexpressed in multiple cancers including early‐stage NSCLC.^111^ A chromosomal translocation involving breakpoints at 11q13.1 leads to the fusion of *MALAT1* with different genes or noncoding regions, such as Alpha‐TFEB, and activation of *MALAT1* in renal cell cancer.^112^ Multiple *MALAT1* fusions have been identified altering the function of the *MALAT1* fusion component (*MALAT1‐SMG*, *EEF1A1‐MALAT1*, *MALAT1‐RFN213*, *MALAT1‐XIST*, *CDK6‐MALAT1*, and *MALAT1‐FAT1*) in neuroendocrine cervix carcinoma,^113^ *MALAT1‐TFEB* fusion in renal cell carcinoma,^112^ and *MALAT1‐AGAT* in mesenchymal liver hemartoma^114^. In addition to fusions, mutations may contribute to overexpression of *MALAT1* in bladder cancer.^115^ *MALAT1* contains a high level of m6A modifications, and its expression can also be upregulated through enhanced stability via m(6)A modification by METTL3,^116^ which alters its structure, mRNA abundance, and alternative splicing.^117^ It indicates multiple activation mechanisms of *MALAT1* in various cancers. *NEAT1* mutations that alter the binding of *NEAT1* to miRNAs have been reported in colorectal cancer patients who relapsed after treatment.^118^ The other alterations observed in lncRNAs are mutations and deletions.

Another common mechanism for the regulation of lncRNA is epigenetically activation or inhibition. LncRNA can be regulated epigenetically, *EPIC1* lncRNA being overexpressed following hypotmethylation and promoting cell‐cycle progression through binding Myc and increasing binding to its downstream target genes in breast cancer^119^ and increasing cell‐cycle progression in breast cancer cell lines.^119^ Hypomethylation of lncRNA *PCAT6* results in overexpression and deregulation of Wnt signalling pathway in LUAD.^120^

### 4.2 Transcriptional regulation of lncRNAs in cancer

LncRNAs are also induced by a variety of physiologic conditions such as hypoxia, hypoxia‐induced noncoding ultraconserved transcripts (e.g., *HINCUT*),^121^ and DNA damage‐induced long‐stress‐induced non‐coding transcripts (e.g., *LSINCT*).^122^ Chemical exposures such as mineral dust, tobacco smoke, and hypoxia upregulate H19.^123^ *HOTAIR* represents one of the most regulated lncRNA in normal and malignant cells. The promoter region of *HOTAIR* contains multiple transcription binding sites (e.g., AP1 and Sp1), interferon‐stimulated response elements,^124^ CArG box,^125^ oestrogen response elements,^96^ and hypoxia response elements,^126^ resulting in regulation by multiple chemicals, therapeutic agents, transcription factors and miRNAs. One of the chemical, bisphenol‐A (used in the production of polycarbonate plastics) and diethylstilbestrol (a synthetic form of oestrogen) interact with oestrogen receptors in the cytoplasm, translocate into the nucleus, and bind to oestrogen receptor elements ERE1 and ERE3 in *HOTAIR*, resulting in its upregulation.^127^ Gefitinib and berberine combination therapies inhibit the expression of *HOTAIR* by inducing miR‐34a‐5p.^128^ In addition, miR‐141 directly binds *HOTAIR* and downregulates its expression in a variety of cancers including renal and prostate cancers.^129^ Interferon regulatory factor 1 (IRF1) downregulates the expression of *HOTAIR* by binding to and interferon‐stimulated response element (ISRE) in its promoter.^124^ MRTF‐A also upregulates the expression of *HOTAIR* by physically interacting with the CArG box in its promoter site.^125^ RNA‐RNA interactions, such as those with heteronuclear ribonucleoproteins (hnRNP A2/B1) can also alter *HOTAIR* transcription.^130^ An SNP (rs920778) in the enhancer of *HOTAIR* induces allelic expression of *HOTAIR* in oesophageal squamous cell carcinoma.^131^

lncRNA are regulated by multiple miRNAs, with for example miR‐512‐5p regulating the *IKBKBAS* lncRNA, promoting cell proliferation, invasion, and metastasis in vitro and increasing metastasis activity in vivo in LUAD. *IKBKBAS* lnc competes with IKKβ for binding to miR‐4741, which leads to overexpression and activation of IKKβ and consequent activation of NF‐κB in LUAD. Interestingly, NF‐κB activation triggered by upregulation of *IKBKBAS* lnc could provide a feed‐forward loop by promoting *IKBKBAS* transcription by binding κB sites within the *IKBKBAS* promoter.^132^ LncRNAs can also be regulated by acetylation. *LINC00467* is upregulated through thymine DNA glycosylase‐mediated acetylation and promotes tumour progression in NSCLC.^133^ In addition to regulation of lncRNAs through different mechanisms, lncRNAs regulate other genes in multiple ways.

### 4.3 LncRNAs control posttranscriptional, translational and posttranslational processes in cancer

lncRNAs, including *MALAT1* and *PLANE*, can alter splicing in other genes that are involved in metastasis. *MALAT1* interacts with the splicing factor SRSF1 and regulates the alternative splicing of BIM, BINI, TEAD and VEGFA pre‐mRNA. It also hijacks SFPQ from the splicing complex SFPQ/PTBP2 and releases the splicing factor PTBP2, altering alternative splicing of cancer‐related isoforms in a variety of cancers.^110^, ^134^ LncRNAs can also directly bind proteins and modulate their functions by regulating the stability of mRNAs or function as host miRNAs by acting as a competitive endogenous RNA, activating signalling pathways and inducing metastasis. ceRNAs can influence cellular functions by: (1) sequestering miRNAs, which bind to miRNAs, preventing them from binding to target mRNAs, or (2) directly binding to mRNA transcripts to block miRNA binding sites.^81^ For instance, *MUC5B‐AS1* modulates metastasis by binding to MUC5B RNA, increasing the stability of MUC5B mRNA by forming an RNA‐RNA duplex.^135^ *JPX* regulates metastasis by acting as a ceRNA for miR‐33a‐5p to control Twist1 expression and mesenchymal‐to‐epithelial transition through Wnt/β‐catenin signalling.^136^ LncRNA can also play an oncogenic role by modulating phosphorylation, such as *SMASR*,^137^ and ubiquitination status of its interaction proteins, such as *PIK3CD‐AS2* ^138^ in lung cancer. LncRNAs can also directly bind proteins in signalling pathways and modulate their functions.

### 4.4 LncRNAs modulate genomic changes in cancer

Deregulated lncRNAs can play a role in the formation of genomic changes and alter genomic integrity. For example, lncRNA *CASTL1* has been shown to stimulate the formation of the *CCDC6‐RET* inversion in radiation or chemical DNA damage‐induced human thyroid cells.^139^, ^140^ LncRNA CCTT has been shown to play a crucial role in centromere function during cell division, specifically in the kinetochore assembly process. CCTT localizes to the centromere through a specific mechanism that involves the formation of an RNA‐DNA triplex and interacts with CENP‐C to recruit it to centromeric DNA during kinetochore assembly. Based on this function, *CCTT* loss leads to mitotic errors and aneuploidy.^141^ *SCAT7* (also known as *ELF3‐AS1*) which is upregulated by DNA‐damaging agents such as cisplatin, promotes cell proliferation. Inhibition of *SCAT7* promotes S phase and G2 block through the ATR pathway, allowing efficient DNA damage repair and homologous recombination.^69^

## 5 The role of lncRNAs in lung metastasis

**FIGURE 4:**
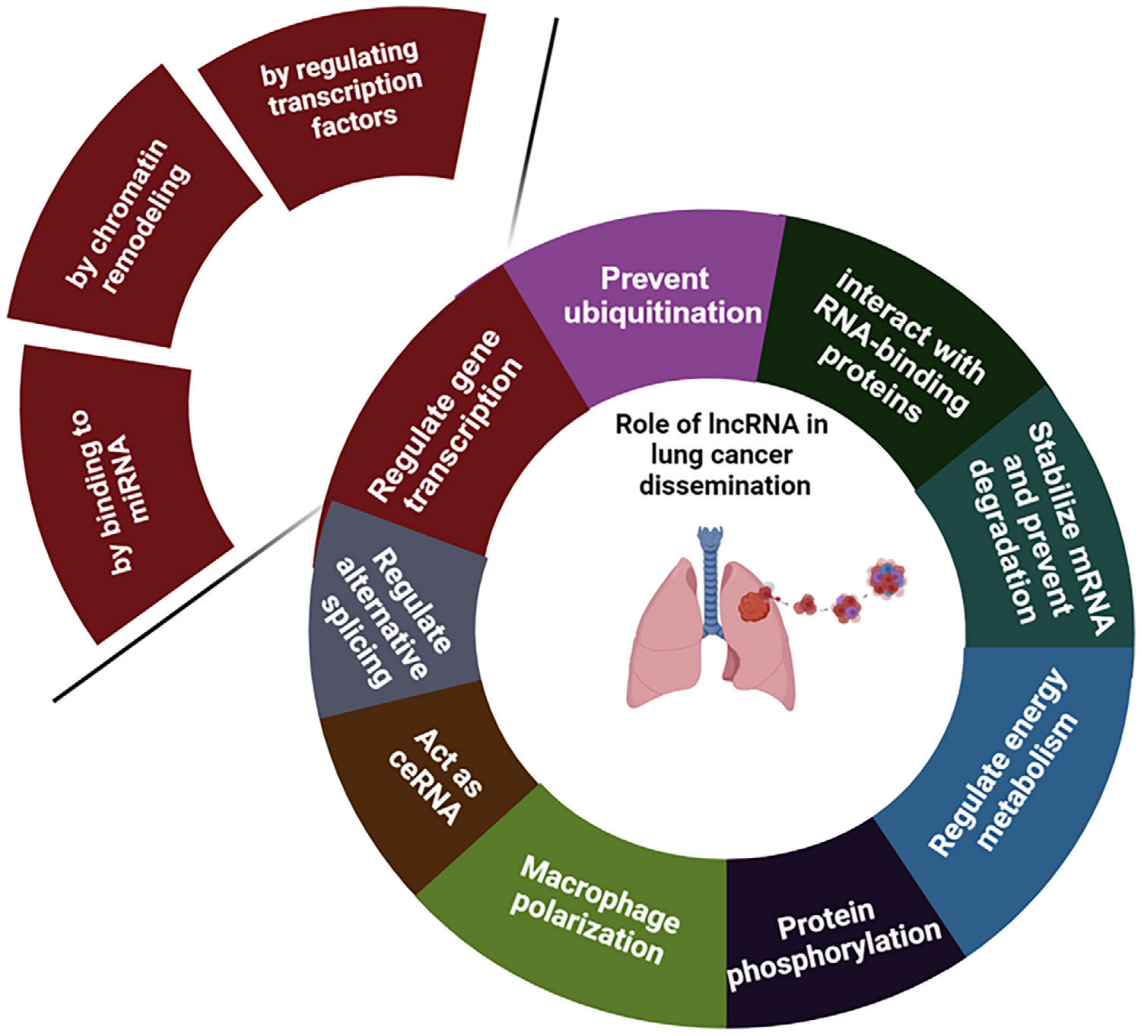
Mechanisms that long non‐coding RNAs (lncRNAs) contribute to lung cancer metastasis. Biorender was used to generate this figure.

As indicated above, lncRNAs are involved in multiple cellular functions such as cell proliferation, autophagy, apoptosis, epithelial‐mesenchymal‐transition (EMT), extracellular matrix (ECM) remodelling, activation of oncogenic signalling pathways, and angiogenesis that contribute to metastasis (Figure 4). lncRNAs are key players in the regulation of metastasis‐related genes. *MALAT1*, or as *Alpha* on chromosome 11q13 was one of the first long non‐coding RNAs (lncRNAs) identified to be associated with cancer. It was originally discovered in lung adenocarcinoma and found to be upregulated in metastatic tumours, which led to its name and its association with cancer metastasis.^111^ Since then, it has been demonstrated to be overexpressed in various other cancers, including breast, liver, colon, and prostate cancer, making it a key player in cancer research. However, the role of *MALAT1* in cancer pathogenesis, progression and dissemination varies by cancer type.

The full‐length *MALAT1* transcript spans approximately 8 kilobases^3^ and exhibits a high degree of sequence conservation across various mammalian genomes. This conservation suggests that *MALAT1* plays a critical, evolutionarily conserved role in cellular functions that are essential for normal development and likely for maintaining tissue homeostasis. The 3′ end of *MALAT1* is subject to modification by specific ribonucleases, such as RNase P and RNase Z, which cleave the RNA to produce a smaller, tRNA‐like non‐coding RNA known as mascRNA (MALAT1‐associated small cytoplasmic RNA).^142^ This processing of *MALAT1* suggests that its various forms, both full‐length (three transcripts) and truncated (one transcript), have distinct functions within the cell. Full‐length *MALAT1* remains predominantly in the nucleus, where it is involved in regulating transcription and splicing events. It specifically localizes to nuclear speckles that serve as sites for the storage, assembly, and modification of various RNA processing machinery, including components involved in splicing and other RNA‐related processes such as heterogeneous nuclear ribonucleoprotein C (HNRNPC), an important splicing factor. *MALAT1* induces a local structural change in pre‐mRNA that increases the accessibility of a U5‐tract, which is a conserved sequence region crucial for the splicing process, facilitating the recognition and binding of HNRNPC. *MALAT1* directly interacts with splicing factor SRSF1 and leads to the formation of mutant p53 R273H and ID4 complex with SRSF1, and consequently relocalization of *MALAT1* from nuclear speckles to chromatin regions that contain H3. This results in the inhibition of the anti‐angiogenic isoform of VEGF and promotes VEGF 121/165 isoforms in breast cancer cells.^143^ *MALAT1* may regulate nuclear speckle scaffold proteins including SON through modulation of other genes.^144^, ^145^ m6A modification on *MALAT1* plays a critical role in regulating cancer cell metastasis in esophageal cancers^146^ linking RNA modification with cancer progression. Specifically, mutant *MALAT1* lacking m6A‐motifs suppresses the metastatic potential of cancer cells, both in vitro and in vivo. Interestingly, *MALAT1* appears to mediate its major functions through altering the expression of many genes involved in oncogenic signalling pathways in lung cancer rather than modulating alternative splicing,^147^ especially in early‐stage lung cancer that eventually metastasize.^111^ *MALAT1* can promote angiogenesis and osteogenic differentiation by regulating SP1/TLR2/BMP2 through direct binding and inhibiting miR‐494 resulting in activation of SP1.^148^

**FIGURE 5:**
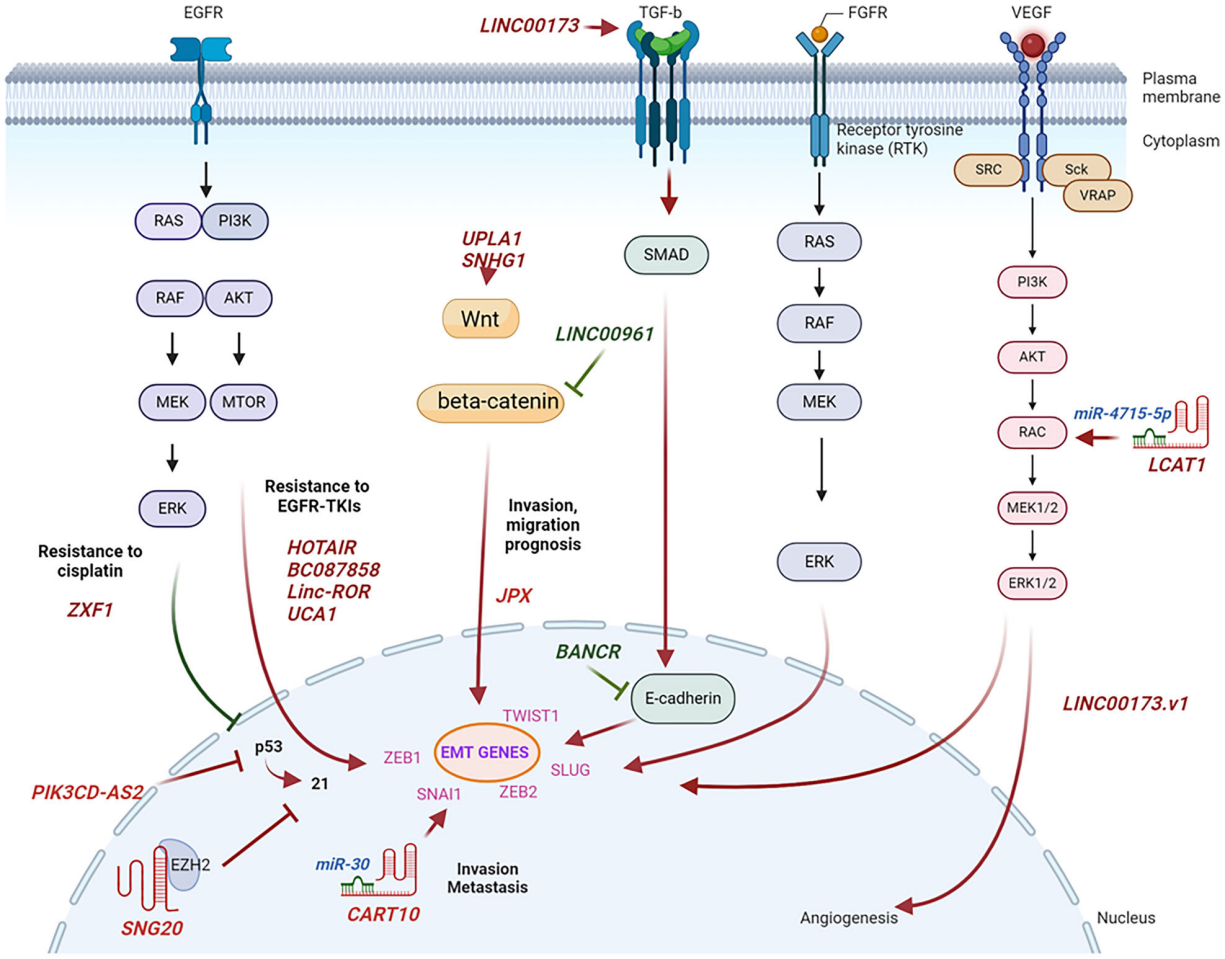
Signalling pathways that long non‐coding RNAs (lncRNAs) involve to lung cancer metastasis. Biorender was used to generate this figure.

Most lncRNAs that have been implicated in lung cancer metastasis can be classified as oncogenic or tumour suppressor based on their function (Tables 2 and 3) and are associated with patient prognosis. LncRNAs have been implicated in altering lung cancer metastasis through multiple different mechanisms (Table 2, Figure 4), including alterations in signalling pathways (Figure 5). These include: (1) recruiting histone modifying enzymes such as EZH2, SUZ12 and LSD1 to epigenetically regulate downstream signalling genes (e.g., *SNGH6*).^149^, ^150^ For example, SNHG6 recruits EZH2 to the promoter region of p27, which leads to elevation of H3K27me3, and consequently to epigenetic inhibition of p27 expression.^108^ LncRNA *FEZF1‐AS1* binds to EZH2 and LSD1 promoters, epigenetically suppressing the expression of E‐cadherin and activating the Wnt/β‐catenin signalling pathway in NSCLC cells.^151^ (2) LncRNAs can act as competing endogenous RNAs (ceRNAs) for miRNA to regulate EMT genes or histone modification proteins and their downstream signalling pathways (e.g., *HOTTIP*) in lung cancer.^152^ Upregulation of the *JPX* lncRNA increases Twist1 expression by acting as a ceRNA and competitively sponging miR‐33a‐5p. Twist1 upregulation induces EMT and lung cancer cell invasion by activating Wnt/β‐catenin signalling.^136^ *LCAT1* that contains miR4715‐5p binding sites also acts as a ceRNA by reducing the ability of miR‐4715‐5p to bind and suppress the expression and function of the Rac family small GTPase 1 (RAC1), leading to upregulation of RAC1 targets in NSCLC.^81^ (3) A number of lncRNAs interact with RNA‐binding proteins (e.g., lncRNA *TINCR* interact with STAU1) to regulate mRNA stability in lung cancer,^153^, ^154^, ^155^ (4) LncRNA have been shown to bind proteins decreasing ubiquitination and degradation or alternatively through altering expression of tumour suppressor genes (e.g., *PIK3CD‐AS2* and *RGMB‐AS1*)^138^, ^156^ or by promoting degradation of proteins with subsequent activation of oncogenic signalling pathways (e.g., *LINC00467*)^133^ or by altering macrophage polarization and immune activation (e.g., *LINC00963* and *SNHG12*),^157^, ^158^ (5) changing protein structure through direct binding (e.g., *LINC00472* and *LINC00525*),^159^, ^160^ (6) bind to miRNA to regulate gene expression (e.g., *CAR10*),^161^ (7) regulate energy metabolism (e.g., *lnc‐IGFBP4‐1*, which is located upstream of IGFBP4),^162^ (8) bind 3′UTR gene regions recruiting ribonucleoproteins to regulate alternative splicing (e.g., *BC009639*),^163^ (9) directly binding to oncogenes and upregulating their expression (e.g., *PD‐L1‐lnc*). *PD‐L1‐lnc* was overexpressed in LUAD independent from *PD‐L1* and, interestingly wild type but not mutated *PD‐L1‐lnc* associates with c‐Myc, enhancing its nuclear translocation and transcriptional activity in LUAD.^164^ (10) LncRNAs physically interact with PRC2 components to regulate expression of other genes (e.g., *FOXM1‐AS*). In lung cancer metastasis, lncRNAs play a significant role in RTK signalling pathways such as transforming growth factor‐β (TGF‐β), EGFR, and VEGF, contribute to metastasis. TGF‐β in late‐stage cancer induces EMT, enhances angiogenesis, promotes cell proliferation, invasion and regulates metastasis.^165^

**TABLE 2:** Long non‐coding RNAs (LncRNAs) are involved in metastasis in lung cancer. All lncRNAs also have function in cell proliferation and/or growth.

Long non‐coding RNA	Tumour subtype	Distant Met/LN	Migration	Invasion	Deregulation mechanisms	Localization	Stage	Prognosis	Cell line study	Tumour tissue	Mice model	Regulated genes and miRNAs	References
Oncogene													
*SNGH6*	NSCLC	na	Y	na	Amplification & Overexpression	na	na	Y	Y	Y	Y	Bind to EZH2 to epigenetically regulates p27	^108^
*SNHG20*	NSCLC	Y/Y	Y	na	Overexpression	N > C	L	Y	Y	Y	Y	Bind to EZH2 to epigenetically regulate p21	^149^
*FEZF1‐AS1* ^*^	NSCLC	/Y	na	Y	Overexpression	N&C	L	Y	Y	Y	na	Bind to LSD1 & EZH2 and epigenetically downregulate E‐cadherin	^151^
*AFAP1‐AS1* ^*^	LUAD but not LUSC	Y/	Y	Y	Overexpression	N&C	L	Y	Y	Y	Y	Bind to SNIP1 in nucleus and stabilize cMyc and inhibit its degradation	^166^
*AFAP1‐AS1*	NSCLC	na	na	na	Overexpression	N > C	L	Y	na	Y	Y	Bind to EZH2 to recruit EZH2 to the promoter regions of p21 and epigenetically regulate p21	^167^
*UFC1* ^*^, ^##^	NSCLC	na	Y	Y	Overexpression	N > C	na	na	Y	Y	Y	Bind to EZH2 to recruit the promoter region of PTEN and repress its expression	^168^
*LINC00963*	NSCLC	Y/Y	Y	Y	Overexpression	N&C	L	Y	Y	Y	Y	Bind to NONO in nucleus and mediate the activity of CRTC. Bind to PKG1 in cytoplasm and prevents its ubiquitination	^169^
*LINC00963*	LUAD	Y	Y	Y	Overexpression	N&C	L	Y	Y	Y	Y	Bind to HNRNPA2B1 protein to regulate stability of Zeb1 by modulating its ubiquitination. Exosomal *LINC00963* induce M2 macrophage polarization	^158^
*LINC01133*	NSCLC	/Y	Y	Y	Overexpression	N	L	Y	Y	Y	Y	Bind to EZH2&LSD1 and downregulate E‐cadherin, KLF2 and p21	^170^
*HOTAIR*	NSCLC	/Y	Y	Y	Overexpression	na	L	Y	Y	Y	Y	Promotes MMP2, MMP9, and inhibits HOXA5 expression	^171^, ^172^
*MALAT1* (*NEAT2*)^**^	LUAD	Y/	Y	na	Overexpression	na	E	Y	Y	Y	Y	Upregulate multiple EMT genes	^111^, ^147^, ^148^, ^173^
*PD‐L1‐lnc* ^&^	LUAD	na	na	Y	Overexpression	N&C	na	na	Y	Y	Y	Bind to cMyc and lead to nuclear translocation and overexpression	^164^
*LINC00173.v1* ^^^^	LUSC	Y/Y	Y	na	Up regulated by delta Np63	C > N	na	Y	Y	Y	Y	Act as a ceRNA for miR‐511‐5p to regulate VEGFA	^174^
*LINC00173* ^&&^	SCLC	na	Y	Y	Overexpression	N > C	L	Y	Y	Y	Y	Act as a ceRNA for miR‐218 to regulates Etk and activates β‐catenin signalling	^175^
*LINC00673* ^*^	NSCLC	/Y	Y	Y	Overexpression	na	na	Y	Y	Y	Y	Act as a ceRNA for miR‐150‐5p to regulate EZH2	^176^
*LINC00673* ^*^	NSCLC	na	Y	Y	Overexpression	na	L	Y	Y	Y	Y	Act as a ceRNA for miR‐150‐5p to regulate ZEB1	^80^
*JPX* ^*^	Lung cancer	Y/	Y	Y	Overexpression	C	L	Y	Y	Y	Y	Act as a ceRNA for miR‐33a‐5p to regulate Twist1 and Wnt/β‐catenin signalling	^136^
*HOTTIP*	SCLC	na	na	na	Overexpression	N&C	L	Y	Y	Y	Y	Act as a ceRNA for miR‐574‐5p to regulate EZH1	^101^
*LCAT1*	NSCLC	Y/	Y	Y	Overexpression	C	na	Y	Y	Y	Y	Act as a ceRNA for miR‐4715‐5p to regulate RAC1	^81^
*LINC01234*	NSCLC	/Y	Y	Y	Overexpression	N&C	L	Y	Y	Y	Y	Act as a ceRNA for miR‐340‐5p & miR‐27b‐3p to bind VAV3 in cytoplasm, and bind to EZH2 and LSD1 and repress expression of BTG2 in nucleus	^177^
*UCA1* ^##^	NSCLC	na	na	na	Overexpression	na	L (III)	Y	Y	Y	na	Act as a ceRNA for miR‐193a‐3p to regulate ERBB4	^178^
*LINC00426* ^*^	LUAD	/Y	Y	Y	Overexpression	C	L	na	Y	Y	Y	Act as a ceRNA for miR‐455‐5p to regulate UBE2V1	^179^
*TTN‐AS1*	LUAD	/Y	Y	Y	Overexpression	na	L	Y	Y	Y	na	Act as a ceRNA for miR‐142‐5p to regulate CDK5	^180^
*LINC00665*	LUAD	/Y	Y	Y	Overexpression	N&C	L	Y	Y	Y	Y	Act as a ceRNA for miR98 to regulate AKR1B10	^155^
*PKMYT1AR*	NSCLC	na	Y	na	Overexpression in tumour and serum. Induced by YY1.na	C > N	na	Y	na	Y	Y	Act as a ceRNA for miR‐485‐5p to regulate PKMYT1 and promotes cancer stem cells maintenance in NSCLC via inhibiting β‐TrCP1 mediated ubiquitin degradation of β‐catenin	^181^
*SOX2OT*	NSCLC	Y/	Y	Y	Overexpression in exosomes	C	na	Y	Y	Y	Y	Act as a ceRNA for miR‐194‐5p to regulate RAC1 expression, and promote bone metastasis	^182^
*IKBKBAS*	LUAD	Y	Y	Y	Overexpression	C > N	E	na	Y	Y	Y	Act as a ceRNA with competing with IKKβ to bind miR‐4741 and results with upregulation of IKKβ mRNA	^132^
*LINC00115*	Lung cancer	Y	Y	Y	Overexpression	na	na	Y		Y	Y	Act as a ceRNA to miR‐607 to regulate ITGB1	^183^
*LINC01342*	NSCLC	/Y	Y	Y	Overexpression	na	E	Y	Y	Y	Y	Binds and reduce the expression of miR‐508‐5p, and miR binds and upregulate expression of CRISP3	^184^
*DLGAP1‐AS1*	NSCLC	na	Y	Y	Overexpression	C	na	na	Y	Y	Y	Binds and reduce expression of hsa‐miR‐193a‐5p and increase DTL expression	^185^
*SNHG1*	NSCLC	/Y	na	na	Overexpression	na	L	Y	Y	Y	Y	Bind to miR‐101‐3p and activate SOX9/Wnt/β‐catenin axis	^186^
*IGFBP4–1* ^#^	Lung cancer	Y/Y	Y	Y	Overexpression	N	L	Y	Y	Y	Y	Regulate energy metabolism	^162^
*CAR10* ^*^	LUAD	/Y	Y	Y	Overexpression	N&C	L	Y	Y	Y	Y	Bind to miR‐30 and miR‐203 and reduce their inhibitory effect on SNAI1 and SNAI2	^161^
*UPLA1*	LUAD	na	Y	Y	Overexpression	N > C	L	Y	Y	Y	Y	It is regulated by YY1 and CAR10 bind to DSP to promote Wnt/β‐catenin signalling	^187^
*LINC00467*	NSCLC	Y/	Y	Y	Overexpression depends on TDG regulated histone acetylation	N&C	L	Y	Y	Y	Y	Bind to AZGP1 and promotes its degradation and activations of Akt pathway	^133^
*PIK3CD‐AS2*	In *TP53* WT LUAD	na	Y	Y	Overexpression	C > N	E	Y	Y	Y	Y	Bind to YBX1^^^ to protects its ubiquitination and degradation, and suppresses p53 signalling	^138^
*CASC9.5* ^*^	LUAD	Y/Y	Y	Y	Overexpression	N > C	L	Y	Y	Y	Y	Bind to DNMT1 and regulate expression of EMT genes	^188^
*MUC5B‐AS1*	LUAD	Y	Y	Y	Overexpression	N&C	L	Y	Y	Y	Y	Bind to MUC5B and increases the stability of MUC5B mRNA by forming a protective RNA‐RNA duplex	^135^
*BC009639* ^^^^^	LUAD	Y/Y	Y	Y	Overexpression	N&C	L	Y	Y	Y	Y	Bind to NCL and 3′UTR of IMPAD to recruit hnRNPD, hnRNPK and IGF2BP1 to regulate alternative splicing of IMPAD1	^163^
Tumour suppressor													
*BANCR* ^*^	NSCLC	/Y	Y	Y	Downregulated	na	L	Y	Y	Y	Y	Regulate E‐cadherin, N‐cadherin and Vimentin expression. HDAC3 downregulates expression of BANCR by Histone deacetylation	^189^
*LHFPL3‐AS2*	NSCLC	Y/Y	Y	Y	Downregulated	N > C	L	Y	Y	Y	Y	Bind to SFPQ and prevents its binding to TXNIP through TGF‐β signalling. Regulated by EGR1 under hypoxia	^190^
*LINC00472* ^*^	LUAD	na	Y	Y	Downregulated	na	L	Y	Y	Y	na	Bind to YBX1 and change the structure and adhesion characteristics, but not change its expression level	^159^
*ZNRD1‐AS1* ^**^	Lung cancer	/Y	Y	na	Downregulated	C > N	na	Y	Y	Y	Y	Act as a ceRNA for miR‐942 to regulate TNS1. YTHDC2 downregulates ZNRD1‐AS1 through m6 A modification	^191^
*SMASR*	Lung cancer	na	Y	Y	Downregulated	N&C	na	Y	Y	Y	N	Bind to SMAD2/3 and downregulates TGFBR1 and also phosphorylates SMAD3. Downregulated by TGF‐β via Smad2/3	^137^
*ADAMTS9‐AS2*	NSCLC	na	Y	Y	Downregulated	na	na	Y	Y	Y	Y	Bind to miR‐223‐3p	^192^
*FOXM1‐AS* ^*^	NSCLC	na	Y	Y	Downregulated	na	na	na	Y	Y	na	Physically interact with PRC2 component EZH2	^193^
*LINC00961*	NSCLC	/Y	Y	Y	Downregulated	na	E	Y	Y	Y	Y	LSD1 bind to *LINC00961* and epigenetically downregulates its expr through H3K4me2 modification. Reduce β‐catenin	^194^
*LINC01186* ^^^	NSCLC	na	Y	Y	Downregulated	N > C	na	na	Y	Y	na	Regulate Snai1, E‐cadherin and vimentin. Regulated by SMAD3	^195^

*Note*: We included lncRNAs that were identified in tumour tissues. LncRNA that were only identified in cell lines and not tested in lung tumour tissues were not included in the Table.

Abbreviations: C, cytoplasm; E, early stage; L, late stage; N, nucleus; na, not available; Y, yes.

^#^ Regulate energy metabolism.

* Regulate epithelial‐to‐mesenchymal transition.

** Regulate angiogenesis.

^##^ Secreted in plasma or serum.

^^^ Regulated by SMAD direct binding to *LINC01186*, BANCR; BRAF activated non‐coding RNA, PD‐L1‐lnc is created by alternative splicing.

^^^^ Promote recurrence.

^^^^^ Lead resistance to EGFR‐TKI.

^&&^ Promote progression and chemoresistance.

**TABLE 3:** Oncogenic and tumour suppressor long non‐coding RNAs (lncRNAs) in lung metastasis.

Oncogenic lncRNAs	Tumour suppressor lncRNAs
*SNGH6, SNHG20, FEZF1‐AS1, UFC1, LINC00963, LINC01133, HOTAIR, MALAT1, PD‐L1‐lnc, LINC00173.v1, LINC00673, JPX, HOTTIP, LCAT1, LINC01234, UCA1, LINC00426, TTN‐AS1, LINC00665, PKMYT1AR, SOX2OT, IKBKBAS, LINC00115, LINC01342, DLGAP1‐AS1, SNHG1, IGFBP4–1, CAR10, UPLA1, LINC00467, PIK3CD‐AS2, CASC9.5, MUC5B‐AS1, BC009639*	*BANCR, LHFPL3‐AS2, LINC00472, ZNRD1‐AS1, SMASR, ADAMTS9‐AS2, FOXM1‐AS, LINC00961, LINC01186*

## 6 LncRNAs in the clinical setting

LncRNAs have the potential to be used for early detection, diagnostic and prognostic biomarkers, to monitor treatment, drug resistance, and in the selection of targeted treatment as well as targets for therapy. This area is only beginning to evolve and enter the clinic.

### 6.1 LncRNAs are involved in chemoresistance in lung cancer

Multiple mechanisms have been proposed to mediate drug resistance in NSCLC. LncRNAs may contribute to acquired resistance to therapy by regulating EMT through receptor tyrosine kinase (RTK) signalling pathways. Further, a number of lncRNAs implicated in metastasis also contribute to resistance to EGFR‐tyrosine kinase inhibitors (TKIs) such as *MALAT1* and *UCA1*, as they are associated with EMT that has been implicated in resistance to multiple drugs (Table 4). *MALAT1* contributes to TKI resistance through activating the p120/miR197‐3p axis.^196^ *UCA1* and *BC087858* both activate the Akt/mTOR pathway and EMT genes in non‐EGFR T790M mutated NSCLC cases leading to drug resistance.^197^, ^198^ *SNHG12* contributes to resistance to cisplatin, paclitaxel and gefitinib by activating the MAPK/slug pathway through miR‐181a in NSCLC.^199^ Overexpression of *MALAT1* also contributes to resistance to cisplatin, adriamycin, gefitinib and paclitaxel by regulating p120 catenin through miR‐197‐3p NSCLC.^196^ *MALAT1* also acts as ceRNA for binding the miR‐146a and miR‐216 to regulate BRCA1 expression, implicating *MALAT1* in DNA damage repair.^200^ Further large scale validation studies and clinical trials are required.

**TABLE 4:** Long non‐coding RNAs (LncRNAs) associated with resistance to therapy in lung cancer.

LncRNA	Regulation	Resistance/sensitivity	References
*SCAT7* (*ELF3‐AS1*)	Up by DNA damaging agents in cisplatin resistant cells	Depletion of SCAT7 leads to decrease cell proliferation in cisplatin resistant cells. Inhibition of SCAT7 increase sensitivity in cisplatin resistance cells	^69^
*UCA1*	Up in EGFR‐TKIs resistance cells	Resistance to EGFR‐TKI, gefitinib by activating AKT/mTOR pathway and EMT in non‐T719M	^197^
*BC087858*	Up	Promote EGFR‐TKIs like gefitinib and erlotinib resistance in non‐T790M mutated samples by activating PI3K/AKT pathway and EMT	^198^
*HOTAIR*	Up	Promote EGFR‐TKIs resistance through EMT Promote resistance to cisplatin. Downregulation of HOTAIR increase sensitivity of NSCLC cells to crizotinib	^201^, ^202^, ^203^
*HOTAIR*	Up	Promote gefitinib resistance through epigenetic regulation of EZH2, and inhibiting p21 and p16	^204^
*Linc‐ROR*	Up in docetaxel resistant cells	Resistant to docetaxel. Blocking linc‐ROR increases docetaxel sensitivity by regulating EMT	^205^
*SNHG12*	Up	Contribute to cisplatin paclitaxel, and gefitinib resistance by activating MAPK/Slug pathway	^199^
*MALAT1*	Up	Contribute to cisplatin, adriamycin, gefitinib, and paclitaxel via regulating the p120/miR‐197‐3p axis. Targeting MALAT1 by ASO reduces metastasis and cell migration in mouse xenograft	^147^, ^196^
MEG3	Down in cisplatin resistant NSCLC cells	Overexpression of MEG3 increases sensitivity to cisplatin by regulating SOX7 through inhibiting miR‐21‐5p	^206^

### 6.2 LncRNAs as therapeutic targets in lung cancer

Most oncogenic lncRNAs involved in lung cancer metastasis are potential therapeutic targets. Integration of pooled CRISPR knockout screening, antisense oligonucleotide inhibitors (ASO), and in vitro functional studies revealed two lncRNAs *CHiLL1* (Cancer Hallmarks in Lung LncRNA 1) and *GCAWKR*, to be associated with poor prognosis. Knockdown of these lncRNAs inhibited cell proliferation and migration in KRAS+ lung cancer cell lines (A549 and H460) and organoids.^207^ Interestingly, *CHiLL1* was mainly localized in the cytoplasm while *GCAWKR* was located in the nucleus. KRAS+ tumours are often treated with cytotoxic platinum‐based chemotherapeutics, but patients usually develop resistance. *CHiLL1* and *GCAWKR* might be alternative targets or modify drug resistance in NSCLC.^207^ Knockdown of *MALAT1*, which, as noted above, is implicated in metastasis of NSCLC, prevents metastasis formation in mouse xenograft model,^147^ is another potential therapeutic target.

LncRNAs with potential oncogenic functions may represent novel therapeutic targets (Table 5). Inhibition of lncRNAs such as *SCAT7* that are overexpressed in the S‐phase in response to DNA‐damaging agents interact with proteins that function to maintain DNA stability, repair, and chromatin remodelling, could prevent or reverse drug resistance. Indeed, inhibition of *SCAT7* decreased cell proliferation in cisplatin‐resistant cells in vitro and in vivo in lung adenocarcinoma.^46^ Inhibition of *LINC01140* reduced tumour growth and metastasis in vivo in the xenograft mice model by inhibiting PD‐L1 through miR‐377‐3p and miR‐155‐5p,^208^ providing another potential target in PD‐L1‐overexpressed lung cancer. Knockdown of *LINC01296* by siRNA in NSCLC in vivo model significantly reduced the tumour mass,^209^ suggesting that siRNA‐based approaches hold potential for targeting lncRNAs in lung cancer therapy.

**TABLE 5:** Long non‐coding RNAs (LncRNAs) that are involved in lung cancer metastasis and are also prognostic markers.

Long non‐coding RNA	Tumour subtype	Prognosis	References
*SNGH6*	NSCLC	Y	^108^
*SNHG20*	NSCLC	Y	^149^
*FEZF1‐AS1*	NSCLC	Y	^151^
*AFAP1‐AS1*	LUAD but not LUSC	Y	^166^
*AFAP1‐AS1*	NSCLC	Y	^167^
*LINC00963*	NSCLC	Y	^169^
*LINC00963*	LUAD	Y	^158^
*LINC01133*	NSCLC	Y	^170^
*HOTAIR*	NSCLC	Y	^171^, ^172^
	LUAD	Y	^111^, ^147^, ^148^, ^173^
*LINC00173.v1*	LUSC	Y	^174^
*LINC00173*	SCLC	Y	^175^
*LINC00673*	NSCLC	Y	^176^
*LINC00673*	NSCLC	Y	^80^
*JPX*	Lung cancer	Y	^136^
*HOTTIP*	SCLC	Y	^101^
*LCAT1*	NSCLC	Y	^81^
*LINC01234*	NSCLC	Y	^177^
*UCA1*	NSCLC	Y	^178^
*TTN‐AS1*	LUAD	Y	^180^
*LINC00665*	LUAD	Y	^155^
*PKMYT1AR*	NSCLC	Y	^181^
*SOX2OT*	NSCLC	Y	^182^
*LINC01342*	NSCLC	Y	^184^
*SNHG1*	NSCLC	Y	^186^
*IGFBP4–1*	Lung cancer	Y	^162^
*CAR10*	LUAD	Y	^161^
*UPLA1*	LUAD	Y	^187^
*LINC00467*	NSCLC	Y	^133^
*PIK3CD‐AS2*	In *TP53* WT LUAD	Y	^138^
*CASC9.5*	LUAD	Y	^188^
*MUC5B‐AS1*	LUAD	Y	^135^
*BC009639*	LUAD	Y	^163^
*BANCR*	NSCLC	Y	^189^
*LHFPL3‐AS2*	NSCLC	Y	^190^
*LINC00472*	LUAD	Y	^159^
*ZNRD1‐AS1*	Lung cancer	Y	^191^
*SMASR*	Lung cancer	Y	^137^
*ADAMTS9‐AS2*	NSCLC	Y	^192^
*LINC00961*	NSCLC	Y	^194^
*RP11‐295g20.2*	LUAD	Y	^55^

As previously mentioned, therapeutic approaches targeting lncRNAs have primarily been explored in preclinical studies aimed at understanding their roles in tumorigenesis and cancer dissemination. These approaches include ASO, CRISPR‐Cas9 editing,^207^ siRNAs,^209^ adeno‐associated virus vectors, small molecules^210^ and nanoparticles.^211^ For example, small molecules such as AC1NOD4Q can disturb the interaction between *HOTAIR* and EZH2 and thereby inhibiting H3K27‐mediated tri‐methylation of nemo‐like kinase without affecting *HOTAIR* expression.^210^ Nanoparticles can be engineered to deliver sRNAs targeting oncogenic lncRNAs or to carry tumour suppressive lncRNAs.^211^ However, none of these strategies have yet progressed to clinical trials specifically targeting lncRNAs in lung cancer. siRNA‐ and ASO‐based approaches have been approved for certain hereditary diseases, but they have been associated with serious side effects, including hepatocellular and renal cytotoxicity.^212^ CRISPR‐Cas9 approaches face challenges such as off‐target effects on neighbouring genes and relatively low editing efficiency, with effectiveness reported at only around 38%.^213^

### 6.3 LncRNAs as prognostic factors in lung cancer

A subset of overexpressed lncRNAs that are implicated in lung cancer metastasis are associated with outcomes (Tables 2 and 5). The association with outcomes suggests that they represent potential prognostic biomarkers. Current preclinical studies are very promising and encouraging to use in clinic. However, currently none of diagnostic and prognostic lncRNAs are used in clinic. Given the tissue specificity of lncRNAs, as well as their subcellular localization, stability and isoforms variability across tissues and organisms, future research will require robust in vitro models such as patient‐derived organoids alongside in vivo studies to elucidate the role of lncRNAs in lung cancer dissemination. Large‐cohort studies and clinical trials will be essential to establish lncRNAs as diagnostic, predictive, and prognostic biomarkers, and to develop them as viable targets for lung cancer therapy.

### 6.4 LncRNAs induced by environmental risk factors

Smoking, air pollution and other environmental factors like arsenic and nickel are risk factors for lung cancer. It is not surprising that lncRNAs are deregulated following exposure to environmental risk factors (Table 6). For example, smoking upregulates the expression of lncRNA *CCAT1*, which inhibits miR‐218 expression, promotes BMI1 transcription, and activates cell cycle progression. *CAR10* expression is higher in lung cancer patients in smoky coal use regions of China than in control regions with an association found between air pollution and *CAR10* overexpression,^214^ CAR10 upregulation induces EGFR overexpression through direct binding and stabilization of YB‐1.^214^ Inhibition of *CAR10* reduces tumour growth in vivo.^214^ PM2.5 induces *LOC146880* expression, invasion, and migration in lung cancer cells,^215^ while arsenic induces *Lnc‐DC* expression and promotes tumour formation through STAT3/ PD‐L1 in arsenic‐transformed cells.^215^ Nickel has been shown to induce hypermethylation of *MEG3* through upregulation of DNMT3b, and promote lung tumorigenesis,^216^ and metastasis through Akt/p70S6K/S6 signalling axis.^217^

**TABLE 6:** Environmental exposure induced long non‐coding RNAs (lncRNAs) in lung cancer.

LncRNAs	Regulation	Environmental exposure	Function	Reference
*CCAT1*	Up	Cigarette smoke	Development and metastasis	^218^
*CAR10*&*YB1*	Up	Dibenzanthracene	Cell proliferation, and upregulate EGFR expression	^214^
*MEG3*	Down	Nickel exposure	Cell proliferation via activating Akt/p70S6K/S6. Downregulation by hypermethylation	^216^, ^217^
*LOC146880*	Up	PM 2.5	Cell autophagy, migration, invasion	^215^
*Lnc‐DC*	Up	rsenic	Involve tumour formation through STAT3/ PD‐L1 in arsenic‐transformed cells	^219^

### 6.5 Exosomal lncRNA

LncRNAs can be secreted from normal cells, tumour cells, tumour‐associated macrophages, and fibroblasts and circulated in body fluids including plasma/serum, sputum, saliva, nasal secretion, urine, semen, breast milk, pleural effusion, tears, amniotic fluid, colostrum, bronchial lavage, cerebrospinal fluid and peritoneal fluid.^220^ Exosomes are endosomal‐derived extracellular small membrane vesicles with lipid bilayers in 40–100 nm diameter that contain various types of bioactive molecules such as nucleic acids (DNAs, mRNAs, noncoding RNAs including lncRNAs), lipids and proteins.^221^ The membrane surface of exosomes is marked by CD9, CD63, CD81, LAMP1, and TSG101, as well as other markers that allow their identification.^222^ Exosomes are a key player in cell‐to‐cell communications and transmit information (e.g., DNA, RNA, proteins and lipids) as cargo to recipient cells.^223^ Exosomal lncRNAs are protected from RNases and can presumably have a longer half‐life than free‐circulating lncRNAs. Thus, exosomes in serum or plasma could serve as readily available samples to evaluate lncRNA levels for evaluation as a biomarker for early detection, monitoring the disease course, or predicting prognosis.

Lung tumour‐derived exosomal lncRNAs can promote metastasis. An example for exosomal lncRNAs includes *SOX2OT* through regulating miRNA‐194‐5p/RAC1 axis in NSCLC,^182^ LncRNA *SOX2OT* (also known as *NCRNA00043* and SOX2 Overlapping Transcript) is overexpressed in plasma exosomes in cases with and without bone metastasis. *SOX2OT* acts as ceRNA for miR‐194‐5p to regulate RAC1. *SOX2OT* contributes to bone metastasis of NSCLC by regulating the TGF‐β/pTHrP/RANKL signalling pathway in osteoclasts through miR‐194‐5p/RAC1 signalling while *MLETA1* promotes metastasis through modulating miR‐186‐5p/EGFR and miR‐497‐5p/IGF1R pathways in small cell lung cancers.^224^ Interestingly, *MALAT1* is significantly higher in plasma from NSCLC patients who have EGFR mutation compared to EGFR WT patients,^225^ suggesting that lncRNA expression, incorporation into exosomes, or release into blood could potentially serve as a diagnostic or predictive biomarker. Exosomal lncRNA *UFC1* is involved in the progression of non‐small cell lung cancer progression.^168^ Suggesting that the deregulation of lncRNAs in tumours, as well as in plasma exosomes, has a regulatory effect on gene expression in multiple cells in the tumour, contributing to metastatic potential. Thus, analysis of lncRNAs in liquid biopsies may identify biomarkers of disease progression. Currently, one clinical trial investigating is exosomal lncRNAs in lung cancer for diagnosis (NCT03830619).

In summary, although these lncRNAs show promise as predictors of chemoresistance, their clinical application is currently limited by challenges in detection methods and the need for extensive large‐scale validation. Further in vivo studies are necessary to fully evaluate their feasibility, specificity, toxicity, effectiveness and to optimize their use in clinical practice.

## 7 CONCLUSION

lncRNAs are crucial regulatory players in genomic stability and cancer development and progression. The large number of lncRNAs in the genome, as well as their diverse functions in cancer and in particular in lung cancer, highlights the importance of whole‐genome sequencing and whole‐cell RNA sequencing to identify aberrations in lncRNA. Moreover, it is important to understand the role of lncRNAs in different tumour cell lineages and clinically relevant subtypes. In addition, it is important to evaluate the contribution of lncRNAs to cancer pathogenesis based on their localization, activation mechanisms and interaction partners. Although changes in lncRNAs are more common in late‐stage tumours, they can be detected in some early‐stage cases. Thus, lncRNAs have the potential to be used for the early detection of lung tumours as well as biomarkers predicting response to therapy and metastatic potential. Moreover, lncRNAs could represent a class of novel therapeutic targets to add to the armamentarium of precision oncology.

## AUTHOR CONTRIBUTIONS

Musaffe Tuna conceived and wrote the paper; Musaffe Tuna, Christopher I. Amos and Gordon B Mills reviewed and edited the manuscript; Christopher I. Amos and Gordon B Mills provided critical suggestions. Christopher I. Amos provided resources. All authors have read and approved the article.

## CONFLICT OF INTEREST STATEMENT

The authors declare no conflicts of interest.

## ETHICS STATEMENT

Not applicable.
